# Bone Regenerative Effect of Injectable Hypoxia Preconditioned Serum-Fibrin (HPS-F) in an Ex Vivo Bone Defect Model

**DOI:** 10.3390/ijms25105315

**Published:** 2024-05-13

**Authors:** Jun Jiang, Lynn Röper, Finja Fuchs, Marc Hanschen, Sandra Failer, Sarah Alageel, Xiaobin Cong, Ulf Dornseifer, Arndt F. Schilling, Hans-Günther Machens, Philipp Moog

**Affiliations:** 1 Experimental Plastic Surgery, Clinic for Plastic, Reconstructive and Hand Surgery, Klinikum Rechts der Isar, Technical University of Munich, D-81675 Munich, Germany; junqing.jiang@mri.tum.de (J.J.); lynn.roeper@mri.tum.de (L.R.); fuchs.finja@gmail.com (F.F.); sarah.alageel@gmail.com (S.A.); xiaobin.cong@tum.de (X.C.); 2 Department of Trauma Surgery, Klinikum Rechts der Isar, Technical University of Munich, D-81675 Munich, Germany; marc.hanschen@mri.tum.de (M.H.); sandra.failer@tum.de (S.F.); 3 Department of Plastic, Reconstructive and Aesthetic Surgery, Isar Klinikum, D-80331 Munich, Germany; ulf.dornseifer@isarklinikum.de; 4 Department of Trauma Surgery, Orthopedics and Plastic Surgery, University Medical Center Göttingen, D-37075 Göttingen, Germany; arndt.schilling@med.uni-goettingen.de

**Keywords:** Hypoxia Preconditioned Serum, injectable fibrin hydrogel, bone defect, osteogenesis, CAM assay, liquid/gas interface model

## Abstract

Biofunctionalized hydrogels are widely used in tissue engineering for bone repair. This study examines the bone regenerative effect of the blood-derived growth factor preparation of Hypoxia Preconditioned Serum (HPS) and its fibrin-hydrogel formulation (HPS-F) on drilled defects in embryonic day 19 chick femurs. Measurements of bone-related growth factors in HPS reveal significant elevations of Osteopontin, Osteoprotegerin, and soluble-RANKL compared with normal serum (NS) but no detection of BMP-2/7 or Osteocalcin. Growth factor releases from HPS-F are measurable for at least 7 days. Culturing drilled femurs organotypically on a liquid/gas interface with HPS media supplementation for 10 days demonstrates a 34.6% increase in bone volume and a 52.02% increase in bone mineral density (BMD) within the defect area, which are significantly higher than NS and a basal-media-control, as determined by microcomputed tomography. HPS-F-injected femur defects implanted on a chorioallantoic membrane (CAM) for 7 days exhibit an increase in bone mass of 123.5% and an increase in BMD of 215.2%, which are significantly higher than normal-serum-fibrin (NS-F) and no treatment. Histology reveals calcification, proteoglycan, and collagen fiber deposition in the defect area of HPS-F-treated femurs. Therefore, HPS-F may offer a promising and accessible therapeutic approach to accelerating bone regeneration by a single injection into the bone defect site.

## 1. Introduction

In the fields of reconstructive plastic and orthopedic surgery, extensive research has been conducted to identify biocompatible methods and materials for the restoration of bone defects [1]. Despite ongoing investigations, the conventional use of autologous bone transplantation persists, albeit accompanied by challenging issues such as limited availability, shaping difficulties, and donor site morbidity [2]. Allografts present an alternative solution, yet their application is constrained by a scarcity of tissue donors [3]. Simultaneously, synthetic implants encounter challenges related to infection susceptibility, unpredictable immunological cell/material interactions, and foreign body reactions [4].

In response to these challenges, significant research has been directed toward implantable bioactive scaffolds, designed to structurally cover defects while serving as carriers for cells and growth factors that actively drive tissue regeneration [5]. Stem cells, particularly mesenchymal stem cells (MSCs) and adipose-derived stem cells (ADSCs), have been explored in conjunction with hydrogels to assess their efficacy in promoting tissue repair [6]. While these approaches exhibit sophistication, they often remain experimental and are subsequently challenged by complex manufacturing processes and high costs [7]. This could indeed explain the growing interest in employing more applicable methods to boost regeneration while utilizing the body’s own resources. Platelet-rich plasma (PRP) is one of the few clinically available autologous treatments for bioactively enhanced tissue regeneration [8]. For bone regenerative therapies, injectable PRP-gel or second-generation platelet-rich fibrin (PRF) have been suggested as innovative blood-derived treatments [9]. While a promising osteogenic effect was found in in vitro experiments, this effect could not be reproduced in several in vivo animal studies and clinical studies [10]. This lack of translation may be attributed to the heterogeneous characteristics of PRP and PRF, particularly in terms of platelet concentration and leukocyte count [9,10].

We previously developed a novel approach using hypoxia-induced protein factors secreted by peripheral blood cells (PBCs), which are incubated extracorporeally at the same physiological temperature and hypoxia typically found in the wound microenvironment [11,12,13,14,15]. These factors were subsequently integrated into injectable fibrin systems to promote tissue regeneration [16]. In particular, our method induces pericellular hypoxia through the oxygen consumption of peripheral blood cells (PBCs) within a closed blood incubation chamber [15,16]. This eliminates the need for environmental hypoxia creation within (expensive) hypoxic incubators, rendering this method more accessible. In our devised system, conditioning peripheral venous blood in a standard syringe at a blood volume per unit cross-sectional area (BVUA) exceeding 1 mL/cm^2^ is adequate for generating sustained pericellular hypoxia (<1% O_2_) [13,14]. The resulting serum compartment, denoted as Hypoxia Preconditioned Serum (HPS), can be extracted and combined with thrombin, calcium, and fibrinogen to form a fibrin gel matrix, referred to as HPS-F [16]. This could provide a platform for a sustained release of growth factors identified in HPS, such as vascular endothelial growth factor (VEGF) [13,15,16,17,18,19], platelet-derived growth factor BB (PDGF-BB) [17,20], basic fibroblast growth factor (bFGF) [17,20], epidermal growth factor (EGF) [18], transforming growth factor-beta1 (TGF-beta1) [17], and insulin-like growth factor-1 (IGF-1) [20]. HPS has been shown to be effective in vitro and in vivo in promoting vascularization [13,15,16,17,18,19,21], fibroblast migration/proliferation [16], and wound healing [16,22,23]. In addition, in vivo studies have highlighted the benefits of growth factor-loaded fibrin matrices (e.g., VEGF), which have exhibited increased local blood vessel ingrowth [24] and integration into recipient tissue through the migration and proliferation of fibroblasts, smooth muscle cells, keratinocytes, and osteoblasts [25]. Thus, utilizing a fibrin matrix carrier for the in vivo administration of HPS growth factors is rational, as it aligns with the matrix employed during native tissue repair [24,25].

**Figure 1:**
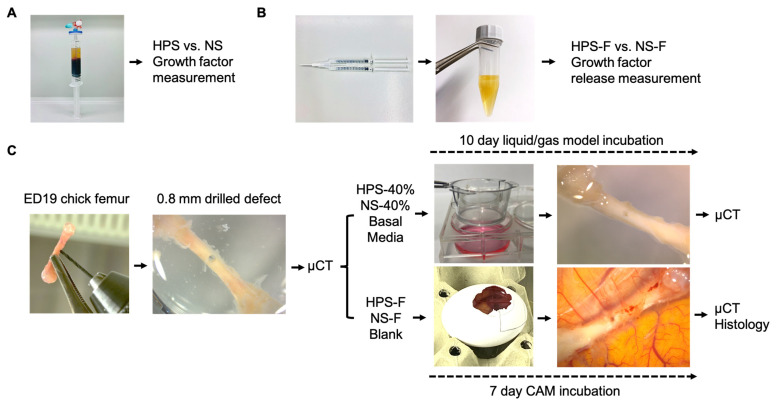
Experimental design. (**A**) Quantitative analysis of bone-related growth factors in HPS (Hypoxia Preconditioned Serum) versus NS (normal serum). (**B**) Production of HPS-F and NS-F by using an interconnected double-syringe (HPS or NS with fibrinogen is combined with thrombin and calcium), followed by measurement of growth factor release over a 7-day period. (**C**) Extracted ED19 chick femur with a precision drill hole of 0.8 mm diameter was analyzed pre-treatment by microcomputed tomography (μCT). Organotypic cultivation included the following: 1. In vitro liquid/gas model with HPS-40%, NS-40%, or basal media supplementation changed every 48 h for 10 days, and 2. in ovo CAM with HPS-F, NS-F single-injection, or no treatment (blank) for 7 days. Post-treatment analysis included μCT and histology.

Since we have previously demonstrated a substantial osteogenic effect of HPS on human osteoblasts in terms of proliferation, migration, and matrix deposition in vitro [26], our current study extends into the field of bone regenerative research utilizing HPS-F in an ex vivo three-dimensional bone defect model. We hypothesize that a single injection of HPS-F can promote osteogenesis and accelerate bone repair. To validate this hypothesis, we initiated our investigation by quantifying bone-related growth factors in HPS and measuring their release from HPS-F over a 7-day period. Subsequently, we first explored the bone regenerative impact of HPS as a medium supplement on drilled bone defects of embryonic day (ED) 19 chick femurs within a liquid/gas interface model. Then, we investigated the effects of HPS-F through a single injection into bone defects using an in ovo chorioallantoic membrane model. Our evaluation focused on microcomputed tomography (μCT) analysis, complemented by histological staining, aimed at assessing structural bone parameters (Figure 1).

## 2. Results

### 2.1. Quantitative Analysis of Bone-Related Growth Factors in HPS

**Figure 2:**
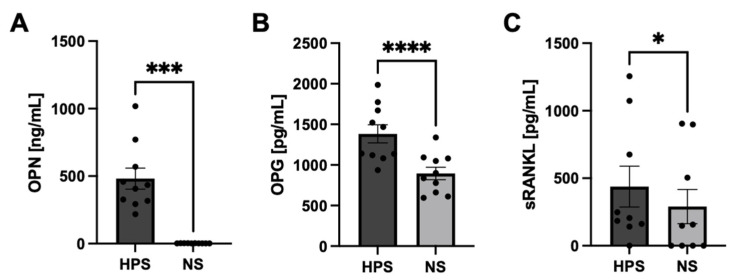
Quantitative analysis of bone-related growth factors in HPS in comparison with NS. (**A**) OPN, (**B**) OPG, and (**C**) sRANKL showed significantly higher concentrations in HPS compared with NS. Paired *t*-test. Data presented as mean ± SEM; blood donors: *n* = 10. * *p* < 0.05, *** *p* < 0.001, and **** *p* < 0.0001.

Continuing the quantitative analysis of growth factors in Hypoxia Preconditioned Serum (HPS) from previous studies that demonstrated elevated concentrations of VEGF, IGF-1, PDGF-BB, TGF-beta1, and bFGF in HPS [15,16,17,20], we conducted measurements encompassing additional bone-related growth factors, including Osteopontin (OPN), Osteoprotegerin (OPG), the soluble Receptor Activator of NF-κB Ligand (sRANKL), bone morphogenetic protein (BMP)-2, BMP-7, and Osteocalcin. Notably, the latter three factors were not detected in either HPS or normal serum (NS). However, in HPS, the concentration of OPN exhibited a remarkable upregulation, reaching levels up to 184 times higher than in NS (481.3 vs. 2.3 ng/mL, *p* < 0.001). Similarly, concentrations of OPG and sRANKL were both 1.5 times higher in HPS compared with NS (1383.0 vs. 894.4 pg/mL, *p* < 0.0001, and 438.2 vs. 290.0 pg/mL, *p* = 0.01, respectively) (Figure 2).

### 2.2. Quantitative Analysis of Growth Factor Release Rates in HPS-F

**Figure 3:**
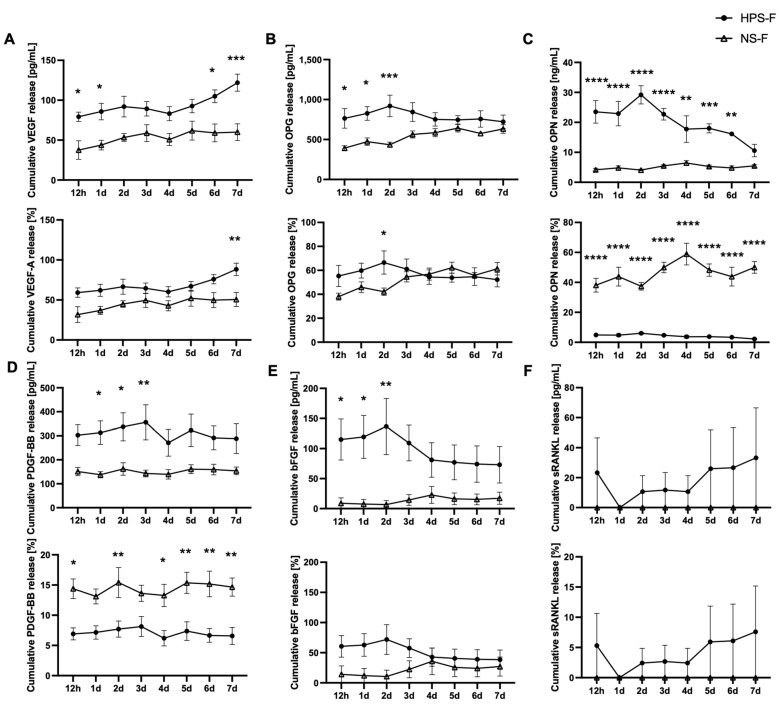
Quantitative analysis of growth factor release rates in HPS-F and NS-F over a 7-day period. Data are provided as cumulative concentrations and as percentages of the initial loading concentration in HPS and NS. (**A**) VEGF-A, (**B**) OPG, (**C**) OPN, (**D**) PDGF-BB, (**E**) bFGF, and (**F**) sRANKL. Data points are means ± SEM; blood donors: *n* = 4. Two-way repeated-measures ANOVA with Sidak’s multiple comparisons test. * *p* < 0.05, ** *p* < 0.01, *** *p* < 0.001, and **** *p* < 0.0001 indicate the statistical comparisons between HPS-F and NS-F from the same timepoints.

For the quantitative analysis of bone-related growth factors released from HPS-F, we measured the cumulative levels of VEGF-A, OPG, OPN, PDGF-BB, bFGF, and sRANKL over a 7-day period and compared the results with the release from NS-F. In addition, we calculated the percentages of the concentrations of released growth factors to the initial loading concentration of HPS and NS (prior to fibrin gelation). For all growth factors, except for sRANKL, the concentrations were significantly higher in HPS-F release than in NS-F release, mostly up to 2–5 times higher within the first 2 days of release (Figure 3A–E). VEGF-A was observed with a sustained release at ~50–60% of the initial VEGF-A concentration in HPS, which increased to 93% on day 7 (Figure 3A). For OPG, the release concentration was also at 50–60% of the initial HPS concentration but sustained throughout the 7-day period (Figure 3B). For OPN, HPS-F release was consistently low at ~1% of the total concentration compared with the 40–50% NS-F release (Figure 3C), indicating a threshold retention capacity of OPN in the fibrin matrix. This effect was very similar to PDGF-BB, where the release of HPS-F was at 5–10%, but NS-F was higher at 15% of the initial concentration (Figure 3D). For bFGF, HPS-F release was also at 50–60% in comparison with the 10–20% NS-F release (Figure 3E). Lastly, the sRANKL release concentration was low at ~5% for HPS-F and was not even detectable in NS-F (Figure 3F).

### 2.3. Microcomputed Tomography Analysis of Femur Defects on a Liquid/Gas Interface Treated with HPS-40% Media

**Figure 4:**
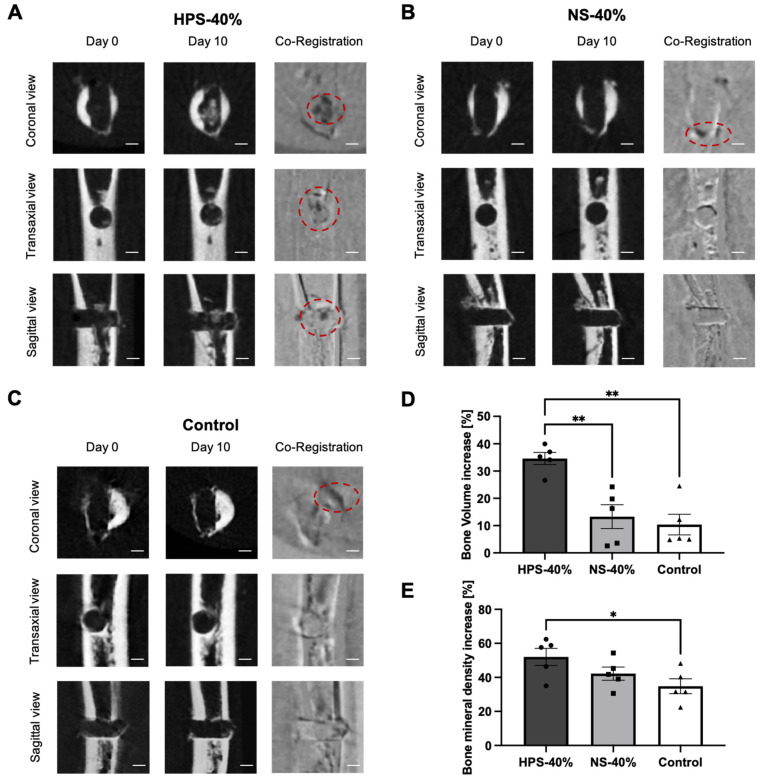
Microcomputed tomography (μCT) analysis of 0.8 mm femur defects on an ex vivo liquid/gas interface treated with HPS-40% compared with NS-40% and (basal media) control. (**A**–**C**) Representative coronal, transaxial, and sagittal views from μCT of day 0 and day 10 and co-registered images of (**A**) HPS-40%, (**B**) NS-40%, and (**C**) control (basal media). Co-registered images display unchanged structures in gray, resorbed structures in white, and new bone formation in black. Red dotted circles indicate the position of new bone formation. Scale bar = 0.5 mm. (**D**) Bone volume increase in the drilled defect area calculated as percentage increase in bone volume/total volume ratio (BV/TV) from day 0 to day 10 of the same volume of interest (VOI). (**E**) Bone mineral density (BMD) increase in the drilled defect area calculated as percentage increase in BMD from day 0 to day 10 of the same VOI. Data presented as mean ± SEM; chick femurs: *n* = 5. One-way ANOVA with Tukey’s multiple comparison test. * *p* < 0.05; ** *p* < 0.01.

To explore the potential bone regenerative effects of HPS, we initially treated 0.8 mm precision drill defects in embryonic day 19 (ED19) chick femurs on a liquid/gas interface with HPS-40% media for a duration of 10 days. The selection of a 40% HPS concentration was based on our previous in vitro study with human osteoblasts, wherein HPS-40% exhibited optimal efficacy in promoting osteoblast proliferation and matrix deposition [26]. The liquid/gas interface assay facilitated the continuous delivery of HPS, achieved through media renewal every 48 h. Our findings revealed a significant increase in bone volume within the defect area by 34.6% in HPS-40% after 10 days (Figure 4A,D). This increase was 2.6 times higher than NS-40% (13.4%; *p* = 0.003) and 3.3 times higher than the control group (basal media) (10.4%; *p* = 0.002), as evidenced by microcomputed tomography (μCT) analysis. In femurs treated with HPS-40%, areas of new bone formation within the defect were identified in the center of the defect using sagittal, axial, and coronal views of the μCT scan (Figure 4A). However, the defect was not completely closed. On the other hand, there was no discernible development of bone mass in the bone defects in the NS-40% and control groups (Figure 4B,C). The bone mineral density (BMD) of the same volume of interest (VOI) revealed an increase of 52.0% in the HPS-40% treated femurs, which was 1.2 times higher than in NS-40% (42.2%; *p* = 0.3) and 1.5 times higher than in the control group (34.8%; *p* = 0.04) (Figure 4E).

### 2.4. Microcomputed Tomography Analysis of HPS-F-Injected Femur Defects in an In Ovo Chorioallantoic Membrane (CAM) Model

**Figure 5:**
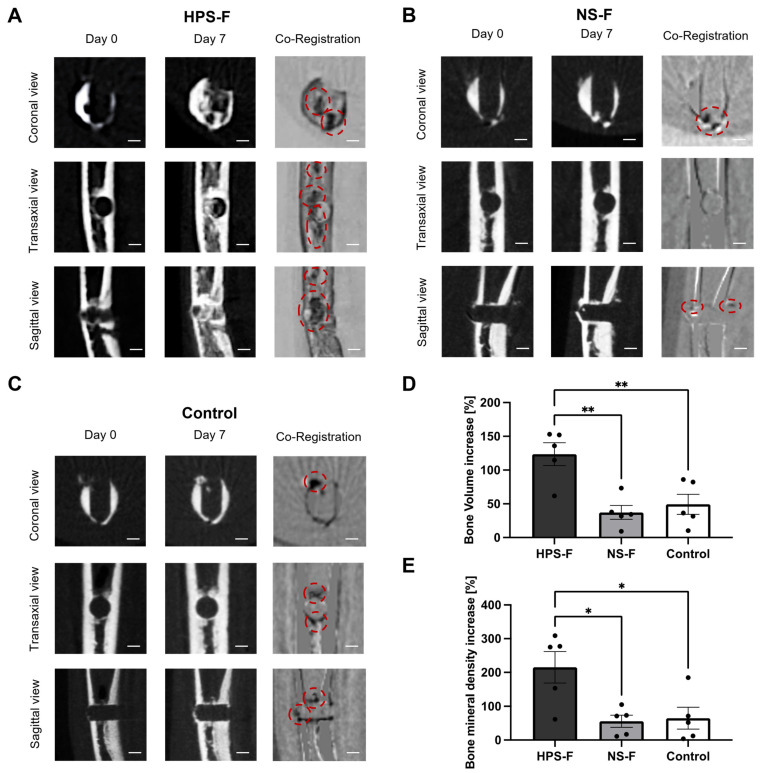
Microcomputed tomography (μCT) analysis of 0.8 mm femur defects implanted on a chorioallantoic membrane (CAM) treated with HPS-F injection compared with NS-F injection and control (no treatment). (**A**–**C**) Representative coronal, transaxial, and sagittal views from μCT of day 0 and day 7 and co-registered images of (**A**) HPS-F, (**B**) NS-F, and (**C**) control (no treatment). Co-registered images display unchanged structures in gray, resorbed structures in white, and new bone formation in black. Red dotted circles indicate the position of new bone formation. Scale bar = 0.5 mm. (**D**) Bone volume increase in the drilled defect area calculated as percentage increase in bone volume/total volume ratio (BV/TV) from day 0 to day 7 of the same volume of interest (VOI). (**E**) Bone mineral density (BMD) increase in the drilled defect area calculated as percentage increase in BMD from day 0 to day 10 of the same VOI. Data presented as mean ± SEM; chick femurs: *n* = 5. One-way ANOVA with Tukey’s multiple comparison test. * *p* < 0.05; ** *p* < 0.01.

Having substantiated the pro-osteogenic effects of HPS media treatment, our subsequent experiment involved the use of Hypoxia Preconditioned Serum Fibrin (HPS-F). In this experiment, HPS-F was injected into 0.8 mm femur defects, which were organotypically cultured in ovo on the chorioallantoic membrane (CAM) of embryonic day 8 (ED8) chicks for a duration of 7 days. The results demonstrated a remarkable 123.5% increase in bone volume, which was 3.3 times higher than NS-F (37.3%; *p* = 0.003) and 2.5 times higher than the control group (no treatment) (49.3%; *p* = 0.008) (Figure 5). An analysis of bone mass production was conducted using sagittal, axial, and coronal views of the μCT. In the femurs treated with HPS-F, areas of new bone formation were observed in the center of the defect that originated from the wall of the drill hole. However, the defect was not completely closed. Moreover, the cancellous bone adjacent to the drill hole exhibited new bone tissue formation, while the overall bone growth of the femur was accelerated, as evidenced by thicker compact bone (Figure 5A). In contrast, the bone defects of the NS-F and no treatment groups did not display substantial bone mass formation in the center of the defect but only at the drilled wall and the defect area at the compact bone level (Figure 5B,C). The bone mineral density (BMD) of the same volume of interest (VOI) corresponded to the bone volume analysis and revealed an increase of 215.2% in the HPS-F treated femurs, which was 3.8 times higher than NS-F (55.5%; *p* = 0.02) and 3.3 times higher than the control group (65.7%; *p* = 0.02) (Figure 5E).

### 2.5. Histological Analysis of Femur Defects

**Figure 6:**
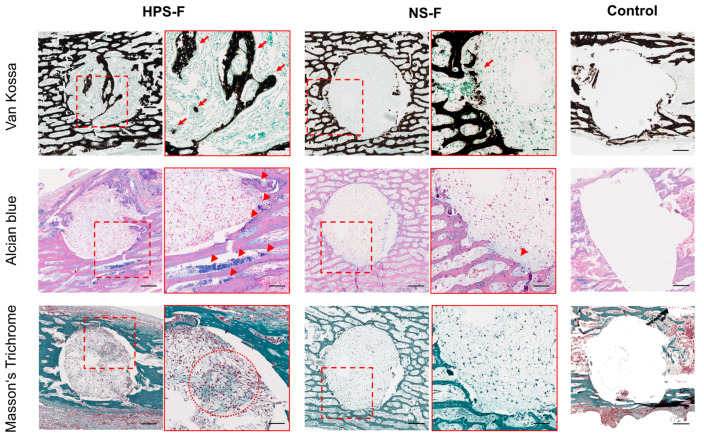
Representative images of histological staining of CAM-cultured femurs treated with HPS-F, NS-F, and no treatment (control) for 7 days. Areas in the red dotted square frames are magnified 2× on the right. Red arrows indicate new calcified bone formation in the Van Kossa staining. Red arrowheads indicate proteoglycan detection in the Alcian blue staining. Red dotted circles show areas of strong detection of collagen in Masson’s trichrome staining. Scale Bar = 0.2 mm, and scale bar in the 2× magnified image = 0.1 mm.

The drilled femurs from the CAM assay were histologically analyzed (Figure 6). Von Kossa staining validated calcified bone tissue in the center of the HPS-F-treated defects (red arrows) in contrast to NS-F and the untreated femurs, confirming previous μCT results. Alcian blue detected proteoglycans predominantly in the area surrounding the defect between the trabeculae, but especially in the HPS-F-treated femurs (red arrowheads). Masson’s trichrome staining revealed collagen fiber invasion into the drilled defect in the HPS-F group (red dotted circle), which was stronger than in the NS-F group. Overall, the area of the drilled defect in the HPS-F treated groups showed a higher density of infiltrating cells than NS-F, while the control group showed almost no tissue and no cells in the center of the defect (Figure 6).

## 3. Discussion

Biomaterials serve as alternative treatments for facilitating bone healing or replacing lost bone tissue by harnessing their properties to induce the recruitment of immature osteogenic cells (osteoinductive) and/or to support bone tissue formation (osteoconductive) [27]. In line with this paradigm, our study focuses on developing a method that delivers osteogenic growth factors while providing a matrix for bone ingrowth. We evaluated the morphological outcome by μCT and histological analysis, which demonstrated the bone regenerative effect of HPS-F administered as an injectable bioactive filler in embryonic day 19 (ED19) chick femur defects. Consequently, HPS-F represents a promising and easily accessible therapeutic approach to accelerating bone regeneration.

In our previous work, we demonstrated elevated levels of TGF-beta1 [20], IGF-1 [20], PDGF-BB [17,20], bFGF [17,20], and VEGF [15,21] in HPS. These biomolecules have been extensively studied for their ability to promote osteogenesis [28]. In this study, we expanded our focus to include Osteopontin (OPN), Osteoprotegerin (OPG), soluble Receptor Activator of NF-κB Ligand (sRANKL), bone morphogenetic protein (BMP)-2, BMP-7, and Osteocalcin as additional quantifiable bone-related growth factors in HPS. Remarkably, BMP-2, BMP-7, and Osteocalcin were undetectable in the secretome of both HPS and normal serum (NS). This observation can likely be attributed to the fact that these growth factors are predominantly produced by osteoblasts rather than peripheral blood cells (PBCs) [29]. Although macrophages have been shown to express BMPs during breast tumor progression and produce microcalcifications in breast tissue [30], the hypoxic preconditioning of PBCs (containing monocytes) did not result in measurable levels of BMP-2 and -7 in HPS. Nevertheless, our study revealed significantly elevated levels of OPN, OPG, and sRANKL in HPS compared with NS (Figure 2). OPN is associated with the promotion of osteoclast migration, adhesion, and activation while also supporting osteoblast development and mineralization, and it is, therefore, involved in bone-remodeling processes [31]. OPG promotes osteogenesis by acting as a decoy receptor for RANKL, thus inhibiting osteoclast activity while promoting osteoblast differentiation and matrix mineralization [32]. On the other hand, RANKL serves as a regulator of bone mass homeostasis by promoting osteoclast formation, maturation, and function [33]. It is a membrane-bound protein on the surface of immune cells, osteoblasts, and various other cells that can be cleaved by matrix metalloproteases and released into the extracellular space as soluble RANKL (sRANKL) [33]. While evidence suggests that osteoclastogenesis requires contact with osteoblasts with membrane-bound RANKL (mRANKL), sRANKL was also able to stimulate osteoclastogenesis from progenitor cells [34]. Interestingly, the subcutaneous injection of sRANKL in mice not only induced high bone turnover and decreased bone volume/density but also increased periosteal bone formation, presumably through osteoblast activation [35,36]. In the context of the OPG/RANKL system, the OPG/RANKL ratio in HPS favors OPG, as sRANKL levels were only detected at very low concentrations (approximately 1000 times lower than OPG). This suggests an overall osteogenic effect of HPS, in addition to the osteogenic growth factors identified in our previous studies mentioned above.

Given the short half-lives of growth factors [37], there is a need to extend their bioactive function by implementing a sustained delivery system. In this regard, it has been shown that fibrin matrices are able to directly bind growth factors via the heparin-binding domain II and release those molecules for at least 7 days [38]. Therefore, the subsequent step in our development process involved fibrin as an HPS factor carrier. This matrix configuration has been explored in previous studies with varying fibrinogen, thrombin, and calcium chloride concentrations [16]. Our observations indicated that the activation of 1 mL of HPS with 18 mg of fibrinogen, 100 IU of thrombin, and 8 µmol of calcium chloride resulted in the highest retention of protein factors within the fibrin matrix and a continuous release from the matrix over 24 h [16]. In this study, we demonstrated a sustained release of VEGF, OPN, OPG, PDGF-BB, and bFGF from HPS-F for up to 7 days. This prolonged delivery opens up the possibility of promoting bone regeneration in the early stages through single-injection therapy. Interestingly, the pro-osteogenic factors VEGF, OPG, and bFGF were released at concentrations of approximately 50% of the loading concentration, which showed previous superior results in osteoblast proliferation, migration, and matrix deposition [26] and in an angiogenesis sprouting assay involving ex vivo aortic rings [15]. The percentage of release for PDGF-BB and OPN was as low as 1–10% of the initial concentration in HPS, indicating a potential retention threshold for these biomolecules within the fibrin matrix. This became more apparent when much lower growth factor concentrations in normal serum (NS) were released in NS-F at a higher percentage of the initial concentration (Figure 3). For the other growth factors, the release rate was proportional to the loading concentrations prior to the fibrin gelation of HPS and NS. Differential retention capacity within the fibrin matrix has been reported previously, which suggests a specific factor-binding capability [13,16,39]. Furthermore, the release kinetics of the growth factors revealed a burst release at 12 h and either a plateau thereafter (OPG, PDGF-BB), a further slight increase (VEGF-A), or a decrease (OPN, bFGF) in concentration toward day 7, indicating a unique relationship between the release of the proteins from the dissolving fibrin matrix and the degradation of the proteins in the release media, as previously reported [13,16]. Interestingly, the concentration of sRANKL was very low in the HPS-F release and even undetectable in the NS release. Considering the anti-osteogenic/regulatory function of sRANKL, this result is favorable for the overall osteogenic effect of HPS-F. When compared with the release of platelet-derived growth factors from PRF (platelet-rich fibrin), studies have shown a similar release profile in growth factors, such as VEGF, PDGF-BB, bFGF, and TGF-beta1, that sustained over 10–14 days [40,41,42,43] but mostly decreased toward 4 weeks [44]. Therefore, this therapeutic efficacy timeframe aligns with the human fracture repair context, where hard callus formation typically begins from the second week of bone healing [45]. Although the effective concentration of growth factors in vivo may be far less due to additional degradation by proteases and hydrolases, it would be interesting to test HPS-F’s release capabilities for up to 4 weeks. This may help us to determine the potential benefit of re-injecting HPS-F at a later point in the bone-healing process.

To validate the defect model of ED19 chick femurs, we initially assessed regenerative effects through HPS media supplementation in a liquid/gas interface model, utilizing an organotypic culture model first described by Dingle and Roach [46]. The choice of the more mature ED19 chick femur was driven by the study’s need to analyze bone regeneration in femurs with higher bone mass (>40%), as opposed to younger ED 7–11 chick femurs with only 5–15% bone tissue and a high percentage of cartilaginous tissue [46]. Although the in vitro organotypic investigation with the liquid/gas interface has been reported to be less effective in bone regeneration than the in ovo CAM cultivation method [46], the supplementation of HPS media at a concentration of 40% demonstrated a notable increase in bone tissue volume within the drilled defect area by up to 34.6% and a bone mineral density (BMD) increase of 52.05%, which was significantly higher than NS-40% and control media supplementation, as demonstrated by μCT analysis (Figure 4). The decision to dilute HPS to 40% was based on earlier findings regarding human osteoblasts, where HPS-40% exhibited optimal concentration for promoting osteogenesis [26]. In alignment with this, the release of pro-osteogenic growth factors in HPS-F, particularly VEGF, OPG, and bFGF, was also found at a similar concentration of approximately 50%. This provided an optimal setting for the subsequent organotypic CAM cultivation. During the CAM cultivation, HPS-F-treated bone defects exhibited a substantial 123.5% increase in new bone formation, which was up to 3.3 times higher than the NS-F and control groups. In addition, BMD increased by 215.2% after HPS-F treatment, which was up to 3.8 times higher than in the other groups, indicating a superior mineralization capacity during bone regeneration, which may lead to increased bone strength at the fracture site. In fact, the promotion of mineralization was previously demonstrated by higher ALP activity and the greater calcification capacity of HPS-stimulated osteoblasts [26]. However, it is noteworthy that HPS-F did not completely close the defect over the 7-day period. Morphologically, we observed bone formation in both the defect region and the surrounding cancellous and compact bone. This was observed to a much lesser extent in the NS-F and control groups (Figure 5). Comparing the results with the literature is challenging since there is a paucity of studies involving ex vivo embryonic chick femur defects [47]. A study using decellularized placenta matrices and human vascular endothelial cell (HUVEC) pellets for ED18 chick femur defects implanted in a CAM for 10 days showed similar results in defect area reduction for a 0.9 mm drilled defect [48]. A 2 mm segmental bone defect in ED18 chick femurs treated with bone marrow mesenchymal stromal cells (BMSCs) in a hydrogel carrier system revealed significant bone formation with a fully closed defect after 8 days of CAM implantation [49]. Considering the complexity of these graft fabrications, HPS-F provides a comparatively simple, cell-free, and accessible method of therapeutic bone repair.

Our histological findings were consistent with the μCT results, revealing calcified bone tissue in the defect area of HPS-F-treated femurs via Van Kossa staining. In terms of soft tissue, we identified a greater amount of cartilaginous tissue around the drilled defect in the HPS-F group using Alcian blue staining for proteoglycan detection. This suggests a potential mechanism of bone formation following HPS-F treatment, possibly involving endochondral ossification. Bone formation via a cartilage intermediate was investigated previously in embryonic chick femurs during physiological fracture repair [50,51] and increased in bone defect regeneration following treatment with BMSCs [49], decellularized matrices [48], and growth factors (e.g., BMP-2, VEGF, and TGF-beta3) [52,53]. Since HPS has previously been demonstrated to have a pro-chondrogenic effect in in vitro chondrocyte cultures, the hypothesis that HPS-F treatment enhances endochondral ossification may be supported [20]. Further investigation into soft tissues has revealed greater collagen production in HPS-F-injected bone defects. Collagen plays a crucial role in providing mechanical support and strength to the bone matrix, acting as a scaffold for bone cells and modulating osteogenesis and osteoblast lineage differentiation through the integrin binding of osteoblast precursors [54]. Future experiments should delve deeper into identifying specific cell types (e.g., progenitor cells, osteoblasts, and osteoclasts), cell activity (e.g., ALP and TRAP) and exploring gene expression profiles related to osteogenic or chondrogenic differentiation (e.g., RUNX2 and SOX9) for a comprehensive understanding of the mechanisms involved.

Finally, the chick femur defect model utilized in this study has limitations that need to be addressed. Although the precision drill defect is placed mid-diaphyseal on the approximately 1 mm thick embryonic femur, slight displacement from the center results in a more cortical defect than other femurs. In addition, small bone fragments were consistently retained in the defect or at the cortical bone level after the drilling process. These confounding factors have also been observed in other studies [48,50] and may result in minimal differences in bone mass formation across the conditions. However, this was not observed to have a considerable effect on the overall percentual calculation of bone mass increase in this study. Furthermore, the regeneration process in embryonic chick femurs relies predominantly on endochondral ossification rather than intramembranous ossification [50]. To address these matters, an in vivo animal experiment using an adult bone defect model that more closely resembles human fracture repair should be included in future experiments to verify the effects of HPS-F, including the analysis of short- and long-term outcomes in bone regeneration and the assessment of biomechanical and microarchitectural properties. Nevertheless, the organotypic model used in this study provides high-throughput analysis results and appears to be adequate for assessing the effect of hormones, growth factors, and other small molecules on bone regeneration.

## 4. Materials and Methods

### 4.1. Production of Hypoxia Preconditioned Serum (HPS)

The production of HPS was based on our previous protocols [16]. Blood donor selection criteria excluded smoking, pregnancy, systemic inflammatory disease, and oral medication intake in the previous 6 weeks. In short, 20 mL of peripheral venous blood was collected under sterile conditions into a 30 mL polypropylene syringe (Omnifix, B. Braun AG, Melsungen, Germany). Then, 5 mL of air was aspirated into the syringe through a 0.2 μm filter (Sterifix, B Braun AG, Melsungen, Germany). The syringe was then sealed to create a closed chamber. The syringes were placed upright in an incubator (37 °C and 5% CO_2_) for 4 days to create pericellular hypoxia of <1% O_2_, as previously described [13,14,16]. Incubation resulted in the separation of the blood into two layers (serum and clot), with the upper layer consisting of HPS. The top layer was filtered into a new syringe to remove cell particles and debris, producing a cell-free product. Depending on the intended use of the HPS, it was either pooled or aliquoted as individual samples. Storage occurred under −80 °C until usage. Samples were stored for a maximum of 3 months.

### 4.2. Production of Normal Serum (NS)

Normal serum was prepared from the same donors as for HPS. Briefly, 20 mL of peripheral venous blood was drawn into a 30 mL polypropylene syringe (Omnifix, B. Braun AG, Melsungen, Germany) and left upright at room temperature for 4 h to enable sedimentation and coagulation of peripheral blood cells. The serum was then filtered through a 0.2 μm filter (Sterifix, B. Braun AG, Melsungen, Germany) and stored at −80 °C until further use for a maximum of 3 months.

### 4.3. Preparation of HPS- and NS-Fibrin (HPS-F and NS-F)

For the preparation of the HPS-F and NS-F, commercially available tissue glue (TISSEEL, Baxter Deutschland GmbH, Unterschleissheim, Germany) was used to form a fibrin gel from HPS and NS. For each 1 mL of HPS or NS, 0.2 mL of fibrinogen (90 mg/mL, TISSEEL, Baxter Deutschland GmbH, Unterschleissheim, Germany) was first mixed with HPS and NS and then activated with 0.2 mL of thrombin and calcium (thrombin: 500 IU/mL; calcium chloride dihydrate: 40 μmol/mL; TISSEEL, Baxter Deutschland GmbH, Unterschleissheim, Germany). The amounts and mix ratios of fibrinogen/thrombin/calcium were adopted from previous studies and showed the highest retention and sustained release capacity [16]. For mixing, we used an interconnected double syringe (Figure 1) with HPS or NS and fibrinogen in one syringe and thrombin and calcium in the other syringe.

### 4.4. Quantification of Bone-Related Growth Factors in HPS and NS

For quantitative measurements of bone-related growth factors in HPS and NS, enzyme-linked immunosorbent assay (ELISA) was performed for Osteopontin (OPN), Osteoprotegerin (OPG), soluble Receptor Activator of NF-κB Ligand (sRANKL), bone morphogenetic protein (BMP)-2, BMP-7, and Osteocalcin according to the manufacturer’s protocol (Osteopontin DuoSet (Catalog #: D1433); Human Trance/ RANK L/ TNFSF11 DuoSet (Catalog #: DY626); Osteoprotegerin/TNFRSF11B DuoSet (Catalog #: DY805); Human BMP-2 DuoSet (Catalog #: DY355); Human BMP-7 DuoSet (Catalog #: DY354); Human Osteocalcin DuoSet (Catalog #: DY1419-05); R&D Systems, Minneapolis, MN, USA)). Readout was performed by optical density measurement using a Mithras LB 940 Multimode Microplate Reader (Berthold Technologies GmbH & Co. KG, Bad Wildbad, Germany).

### 4.5. Quantification of HPS-F and NS-F Growth Factor Release

The release of bone-related growth factors from HPS-F and NS-F was evaluated over 7 days. The growth factors examined included vascular endothelial growth factor-A (VEGF-A), OPG, OPN, platelet-derived growth factor BB (PDGF-BB), basic fibroblast growth factor (bFGF), and sRANKL. For the experimental setup, HPS-F and NS-F were prepared as described in Section 4.3 for 1 mL of HPS and NS, respectively, and transferred into a 5 mL tube (Eppendorf, Hamburg, Germany). After a gelation time of 1 min, 1 mL of PBS was added, and the tubes were incubated on a shaker plate at room temperature. The supernatants were collected after 12 h and after 1, 2, 3, 4, 5, 6, and 7 days. Samples were stored at −80 °C until ELISA measurements were taken. ELISAs were carried out according to the manufacturing protocol (VEGF DuoSet ELISA (Catalog # DY293B); OPG DuoSet ELISA (Catalog # DY805); OPN DuoSet ELISA (Catalog #: DY1433) or PDGF-BB DuoSet ELISA (Catalog #: DY220); bFGF DuoSet ELISA (Catalog #: DY233) R&D Systems, Minneapolis, MN, USA). Readout was performed through measurement of the optical density using a Mithras LB 940 Multimode Microplate Reader (Berthold Technologies GmbH & Co. KG, Bad Wildbad, Germany).

### 4.6. Femur Defect Preparation

Fertilized chick eggs were purchased from VALO (BioMedia GmbH, Osterholz-Scharmbeck, Germany) and placed in an egg incubator (Breeding Machine 48 Eggs incubator, Wiltec, Eschweiler, Germany) with a constant temperature of 36.6 °C, with a humidity of 60%, and in rotation mode until embryonic day (ED) 19. The eggs were then opened, and the embryo was euthanized by decapitation. Both femurs were surgically dissected and placed in PBS supplemented with 1% antibiotic/antimycotic solution (Capricorn Scientific GmbH, Ebsdorfergrund, Germany) for 10 min at room temperature. Then, a standardized, precision drill defect was placed in the center of the diaphysis with a 0.8 mm drill (Dremel 4000 multitool system, Dremel, Racine, WI, USA). The femurs were scanned by microcomputed tomography prior to the organotypic cultivation (see Section 4.9).

### 4.7. Ex Vivo Liquid/Gas Interface Cultivation of Drilled Chick Femurs

For the organotypic cultivation of drilled chick femurs with HPS and NS media supplementation, a liquid/gas interface assay was performed, as described by Dingle and Roach [46]. Each bone was placed on a 0.4 µm pore size cell insert (FALCON, Corning Incorporated, Durham, NC, USA) in a 6-well plate (Greiner CELLSTAR, Frickenhausen, Germany) with 1 mL of treatment or control media. HPS-40% and NS-40% treatment media were prepared by mixing 400 µL of HPS or NS with 600 µL of culture medium containing DMEM (PAN BIOTECH, Aidenbach, Germany), 10% FCS (Biochrom GmbH, Berlin, Germany), 1% antibiotic/antimycotic solution (Capricorn Scientific GmbH, Ebsdorfergrund, Germany), and 100 µmol of ascorbic acid (Merck, Darmstadt, Germany). The control group consisted of a 1 mL culture medium. The plates were incubated at 37 °C and 5% CO_2_ for 10 days, and media were changed every 48 h. After 10 days, a microcomputed tomography scan was performed (see Section 4.9).

### 4.8. In Ovo CAM (Chorioallantoic Membrane) Cultivation of Drilled Chick Femurs

For the organotypic cultivation of drilled chick femurs with the HPS-F and NS-F treatments, an in ovo CAM assay was performed. Fertilized chick eggs were purchased from VALO (BioMedia GmbH, Osterholz-Scharmbeck, Germany) and were kept at 15–18 °C upon arrival for at least 24 h and up to 7 days to ensure the calming of the embryos followed by slow warming to room temperature within 12 h. Then, the eggs were placed in an egg incubator (Breeding Machine 48 Eggs incubator, Wiltec, Eschweiler, Germany) with a constant temperature of 36.6 °C, with a humidity of 60%, and in rotation mode. On ED7, the eggs were opened for the CAM assay by creating a round hole in the eggshell, which was sealed with a transparent adhesive film (3M Tegaderm, Fresenius Kabi GmbH, Bad Homburg, Germany) and returned to the egg incubator for another 24 h until the bones were implanted. On the day of implantation, the defects in the bones were injected either with 0.2 mL of HPS-F or NS-F using the interconnected double syringe or received no treatment (blank) in the control group and were placed on the CAM. The eggs were sealed with transparent adhesive film and incubated for 7 days in the egg incubator using the same parameters as described above, with rotation mode turned off. Prior to explantation, 300 µL of 3.7% formaldehyde (Otto Fischer, Saarbruecken, Germany) was applied to the CAM for fixation. The bone was harvested with a piece of the CAM, and the chick embryo was euthanized by decapitation. The bone was first analyzed by microcomputed tomography and then by histology (see Section 4.9 and Section 4.10).

### 4.9. Microcomputed Tomography Scan

The microcomputed tomography (µCT) scan was performed before and after the organotypic cultivation using a SkyScan 1176 (Bruker Corporation, Kontich, Belgium). Each bone was placed in a 1.5 mL tube (Eppendorf, Hamburg, Germany) filled with PBS to remove the air surrounding the bone tissue and additionally stabilized with sterile gauze. The samples were scanned at a pixel size of 33 µm, employing an aluminum filter of 0.2 mm and an X-ray tube voltage of 45 kV with a rotation interval of 0.7°. Reconstruction was carried out with a modified Feldkamp algorithm using the SkyScan NRecon software (version 1.7.4.2, Bruker Corporation, Kontich, Belgium). Gaussian smoothing (1), ring artifact reduction (5), and beam-hardening correction (25%) were applied identically for all samples. Misalignments were corrected individually. The same volume of interest (VOI) was defined over the drill hole, including the drill wall, in all three axes. The respective reconstructed data sets of the same femur, before and after implantation, were aligned in the same position in all three planes using the Data-Viewer software (version 1.5.6, Bruker Corporation, Kontich, Belgium) for a standardized analysis. The increase in bone volume was calculated as the percentage of the bone volume/total volume ratio at the beginning to the ratio at the endpoint of cultivation using the CTAn software (version 1.20.3.0+, Bruker Corporation, Kontich, Belgium). To calculate the bone mineral density (BMD), a 4 μm calibration rod pair was scanned under the same conditions as the femurs. The phantoms were composed of epoxy resin with embedded fine calciumhydroxyapatite at concentrations of 0.25 g/cm^3^ and 0.75 g/cm^3^. The increase in BMD was calculated as the percentage of the ratio of the BMD at the beginning to the BMD at the endpoint of cultivation. The analysis was performed semi-automatically using BatMan in CTAn. Global thresholds were selected by visual matching with grayscale images.

### 4.10. Histology

The femurs were fixed in 3.7% formaldehyde for 5 days at 4 °C. The samples were dehydrated through a graded series of ethanol, embedded in paraffin; serially sectioned at 5 μm thickness from the center; examined on a coated glass slide; and stained with H&E, Van Kossa, Alcian blue, and Masson-Goldner’s trichrome. A Leica Aperio microscope (Leica Biosystems, Nussloch, Germany) was used to digitally scan the slides.

### 4.11. Statistical Analysis

Data sets were analyzed by a paired *t*-test if two comparison groups were available. If more than two groups and one independent variable were present, a one-way analysis of variance (ANOVA) with subsequent comparisons using Tukey’s post hoc analysis was performed. If two independent variables were present, repeated-measures two-way analysis of variance (RM-ANOVA), with subsequent comparisons using Sidak’s post hoc analysis, was performed. All values are expressed as means ± standard error of the mean (SEM). A value of *p* < 0.05 was considered statistically significant (* *p* < 0.05, ** *p* < 0.01, *** *p* < 0.001, and **** *p* < 0.0001).

## 5. Conclusions

This study introduces a novel approach involving the incorporation of hypoxia-induced growth factors into a fibrin matrix as a bioactive filler for the enhancement of bone repair in an ex vivo bone defect model. The results highlight the efficacy of the hypoxia preconditioning of PBCs and the sustained delivery of their secretome from a fibrin matrix as a strategy to induce osteogenesis. Consequently, HPS-F holds the potential to regenerate bone defects, particularly in scenarios, such as acute fractures or delayed union, through a singular injection. Further in vivo experiments are needed to verify its effect in a clinical setting.

## 6. Patents

Device-based methods for localized delivery of cell-free carriers with stress-induced cellular factors (AU2013214187 (B2); 9 February 2017): Schilling Arndt, Hadjipanayi Ektoras, and Machens Hans-Günther.

## Data Availability

The data that support the findings of this study are available from the corresponding authors upon reasonable request.
